# Adaptation and initial psychometric validation of the Latvian version of EORTC QLQ-C30 and QLQ-BR45 in women with breast cancer

**DOI:** 10.1007/s11136-026-04394-7

**Published:** 2026-09-25

**Authors:** Ingrida Trups-Kalne, Laura Nesaule, Lauma Springe, Lubova Tihomirova, Vita Steina, Viktorija Pacukevica-Stope, Laura Rotberga, Jelena Maksimenko, Arvids Irmejs, Elita Poplavska

**Affiliations:** 1 Psychology Laboratory, Institute of Public Health, Rīga Stradiņš University, Riga, Latvia; 2 Institute of Public Health, Rīga Stradiņš University, Riga, Latvia; 3 Institute of Public Health, Faculty of Social Sciences, Rīga Stradiņš University, Riga, Latvia; 4 Institute of Oncology and Molecular Genetics, Rīga Stradiņš University, Riga, Latvia; 5 Breast Unit, Pauls Stradiņš Clinical University Hospital, Riga, Latvia; 6 Breast Unit, Department of Surgery, Pauls Stradiņš Clinical University Hospital, Riga, Latvia; 7 Department of Pharmacology and Pharmacotherapy, Institute of Public Health, Faculty of Pharmacy, Rīga Stradiņš University, Riga, Latvia

**Keywords:** Patient-reported outcome measures, Breast cancer, Health-related quality of life, EORTC QLQ-C30, EORTC QLQ-BR45, Validation

## Abstract

**Purpose:**

To culturally adapt and initially evaluate the psychometric properties of the Latvian versions of the EORTC QLQ-C30 and breast cancer-specific EORTC QLQ-BR45 in women with breast cancer.

**Methods:**

The study was conducted within a pilot project implementing the ICHOM Breast Cancer Standard Set in a specialised university hospital in Latvia. Translation and cultural adaptation followed the EORTC procedure and included expert review and cognitive debriefing. Psychometric evaluation used baseline data from 142 women and six-month follow-up data from 47. Analyses included item-level indicators, internal consistency, multitrait scaling, inter-scale correlations, correlations with the Stress Symptom Scale, and paired change analyses. Conditional QLQ-BR45 items were treated as structurally missing when not applicable.

**Results:**

Expert review and cognitive debriefing supported linguistic, conceptual, and cultural appropriateness. Reliability and item-scale correspondence were acceptable for several domains, with particularly strong multitrait scaling results for QLQ-BR45 Body Image, Sexual Functioning, and Breast Symptoms. Correlations between EORTC functioning and symptom domains and the Stress Symptom Scale were generally in the hypothesised directions. Six-month findings showed clinically plausible changes in functioning and treatment-related symptoms, but estimates were based on a smaller follow-up sample and reduced valid samples for some conditional scales.

**Conclusion:**

The Latvian EORTC QLQ-C30 and QLQ-BR45 showed acceptable preliminary psychometric performance. The findings provide initial support for their use to assess HRQoL and breast cancer-specific symptoms in Latvia, while larger multicentre and longitudinal studies are needed to evaluate additional measurement properties and responsiveness.

## Abstract (plain language summary)

This study evaluated Latvian versions of two questionnaires used internationally to understand quality of life and symptoms in women with breast cancer: the EORTC QLQ-C30 and QLQ-BR45. Latvia lacked a comprehensive evaluation of these versions in women receiving breast cancer care. The translations were reviewed by specialists and tested with patients to ensure that the wording was understandable and culturally appropriate.

We then analysed questionnaire data from 142 women before treatment and from 47 women six months later. Several questionnaire scales showed acceptable reliability and the expected relationships with related symptoms. The follow-up results also showed plausible changes during treatment, although the smaller follow-up group means these findings are preliminary. Overall, the Latvian questionnaires appear suitable for cautious use in research and clinical practice, but larger studies are needed to confirm how well they perform across different patient and treatment groups.

## Introduction

Breast cancer is the most frequently diagnosed malignancy among women worldwide and remains a major public health concern. In Latvia, 1,335 new breast cancer cases were diagnosed in women in 2024 [1]. Health-related quality of life (HRQoL) complements clinical outcomes by capturing the physical, psychological, and social effects of cancer and its treatment, including fatigue, body-image concerns, and treatment-related symptoms [2, 3].

The European Organisation for Research and Treatment of Cancer (EORTC) QLQ-C30 is a widely used core cancer HRQoL questionnaire, supplemented by disease-specific modules [4]. The QLQ-BR45 extended the earlier breast cancer module to reflect contemporary treatments and associated patient-reported outcomes [5]. Three items were subsequently removed during final validation, resulting in the QLQ-BR42; however, the QLQ-BR42 was published after the present project had begun, so the QLQ-BR45 was used in this study [5, 6].

Cross-cultural use of patient-reported outcome measures requires careful translation and evaluation to preserve conceptual equivalence and measurement properties, particularly for culturally sensitive domains such as sexuality and body image. Although Latvian translations of EORTC instruments are needed for clinical and research use, comprehensive psychometric evaluation of the Latvian QLQ-C30 and QLQ-BR45 in women with breast cancer has been lacking.

This study therefore aimed to culturally adapt and initially evaluate the psychometric properties of the Latvian versions of the EORTC QLQ-C30 and QLQ-BR45 in women with breast cancer.

## Method

The study was conducted within a pilot project implementing the ICHOM Breast Cancer Standard Set at Pauls Stradiņš Clinical University Hospital, Latvia. Patient-reported outcomes were collected electronically before treatment (T1) and at six (T2) and 12 months (T3); the present analysis used T1 and T2 data. Adaptation of the QLQ-BR45 followed the EORTC Quality of Life Group (QLG) Translation Procedure [7, 8].

Translation sought conceptual and linguistic equivalence while preserving the original item content, order, response options, and scoring structure [7, 9].

### Translation and cultural adaptation

After permission from the EORTC QLG, two independent forward translations into Latvian were reconciled by the translators with input from six clinicians and a psychometrics specialist. The expert review assessed linguistic accuracy, conceptual equivalence, cultural appropriateness, and comprehensibility; it was not intended to re-evaluate the content validity of the original EORTC instruments. Expert comments were used only for translation-level refinement.

Two independent back-translations were then prepared by native English speakers fluent in Latvian and blinded to the original questionnaire. Discrepancies were reviewed against the source version, and translation decisions and revised materials were submitted to the EORTC QLG for approval.

### Cognitive debriefing

Ten Latvian-speaking breast cancer survivors with experience of different treatments completed the Latvian QLQ-C30 and QLQ-BR45 and participated in structured interviews on item clarity, acceptability, cultural appropriateness, and response options [7]. Minor linguistic refinements were documented and submitted to the EORTC QLG; underlying concepts and scoring were unchanged.

### Psychometric evaluation

The psychometric evaluation followed EORTC guidance and scoring procedures [8, 10, 11]. Analyses were selected to match the available sample and included item-level performance, internal consistency, multitrait scaling, correlation-based construct validity, and preliminary change over time. Confirmatory factor analysis and measurement invariance testing were not conducted because the sample was insufficient for stable model-based analyses.

## Participants

Phase 1 included six clinical experts: two surgical oncologists, two general practitioners, one nurse, and one occupational therapist.

Phase 2 included ten Latvian-speaking breast cancer survivors in remission who had experienced a range of treatments, including surgery, systemic therapy, radiotherapy, endocrine therapy, reconstruction, and prophylactic procedures.

Phase 3 included women aged 18 years or older with a first-time breast cancer diagnosis, sufficient Latvian proficiency, and treatment at the Breast Cancer Centre of Pauls Stradiņš Clinical University Hospital. Participants provided informed consent before accessing the electronic platform. Recruitment occurred from November 2024 to February 2026; 142 women completed baseline assessment and 47 completed the six-month follow-up.

## Data collection and instruments

Baseline data included demographic characteristics, information received about treatment options, self-reported health characteristics and comorbidities, and clinical disease and treatment information extracted from electronic health records.

The EORTC QLQ-C30 is a 30-item cancer-specific HRQoL questionnaire comprising global health/QoL, five functioning scales (Tables 1 and 2), three multi-item symptom scales, and six single-item symptoms/problems [12, 13].

**Table 1:** Structure of the EORTC QLQ-C30 [13]

	Scale	Number of items (*n*)	Item range*	Item numbers
Global health status/QoL				
Global health status/QoL	QL2	2	6	29, 30
Functional scales				
Physical functioning	PF2	5	3	1–5
Role functioning	RF2	2	3	6–7
Emotional functioning	EF	4	3	21–24
Cognitive functioning	CF	2	3	20, 25
Social functioning	SF	2	3	26–27
Symptom scales/items				
Fatigue	FA	3	3	10, 12, 18
Nausea and vomiting	NV	2	3	14–15
Pain	PA	2	3	9, 19
Dyspnoea	DY	1	3	8
Insomnia	SL	1	3	11
Appetite loss	AP	1	3	13
Constipation	CO	1	3	16
Diarrhoea	DI	1	3	17
Financial difficulties	FI	1	3	28

*Item range = maximum minus minimum response. Items 1–28 use 1–4 responses (range = 3); items 29–30 use 1–7 responses (range = 6)

QLQ-C30 scores were transformed to 0–100 scales according to the EORTC scoring manual; higher functioning/global health scores indicate better status, whereas higher symptom scores indicate greater symptom burden [11, 13].

The EORTC QLQ-BR45 is a 45-item breast cancer-specific module used with the QLQ-C30. It assesses body image, future perspective, sexuality, and breast cancer- and treatment-related symptoms [5, 9]. Scores were transformed to 0–100 using EORTC procedures.

Most QLQ-BR45 items use four response categories. Sexual and endocrine-sexual items refer to the previous four weeks. Sexual Enjoyment and Endocrine Sexual Symptoms are conditional on sexual activity, and Upset by Hair Loss is conditional on reported hair loss; non-applicable responses were treated as structurally missing rather than as absence of symptoms.

**Table 2:** Structure of the EORTC QLQ-BR45 [9]

	Scale	Number of items (*n*)	Item range*	Item numbers
Functional scales				
Body Image	BI	4	3	39–42
Future Perspective	FU	1	3	43
Sexual Functioning	SX	2	3	44–45
Sexual Enjoyment	SE	1	3	46
Symptom scales				
Systemic Therapy Side Effects	SYS	7	3	31–34, 36–38
Upset by Hair Loss	HU	1	3	35
Arm Symptoms	ARM	3	3	47–49
Breast Symptoms	BR	4	3	50–53
Endocrine Therapy Symptoms	ET	10	3	54–56, 63–69
Skin Mucosis Symptoms	SM	6	3	57–62
Endocrine Sexual Symptoms	ES	4	3	70–73

*“Item range” is the difference between the possible maximum and the minimum response to individual items. All items are scored 1 to 4, giving range = 3

The 56-item Stress Symptom Scale includes physical, cognitive, psychological, and emotional symptom domains rated from 0 (almost never/never) to 5 (almost all the time/continuously) [14]. It was used as a related external comparison measure, not as a criterion measure of cancer-specific HRQoL, because validated Latvian-language comparison measures are limited and several stress domains overlap conceptually with EORTC functioning and symptom domains.

### Statistical analysis

Scale scores were calculated according to EORTC rules. Multi-item scale scores were computed when at least half of the constituent items had valid responses; otherwise the score was missing. Conditional QLQ-BR45 items/scales were structurally missing when not applicable. Reliability analyses used valid scale-level cases, and correlations used pairwise deletion; valid N therefore varied across conditional scales and coefficients.

Analyses were conducted in Microsoft Excel and IBM SPSS 29. Descriptive statistics and corrected item–total correlations were calculated. Internal consistency was assessed with Cronbach’s alpha for multi-item scales and McDonald’s omega where applicable [15–17]. Multitrait scaling used corrected item–own scale correlations for convergence and item–other scale correlations for distinctiveness; convergent success required item–own *r* ≥ .40, and scaling success required item–own r to exceed the highest absolute item–other scale correlation. Spearman correlations assessed relationships between EORTC scales and with the Stress Symptom Scale. Functioning scales were hypothesised to correlate negatively with stress scores and symptom scales positively. Paired T1–T2 changes were tested with Wilcoxon signed-rank tests using participants with valid scores at both time points and were interpreted as preliminary evidence of change rather than definitive responsiveness. Expert agreement indices used the CVI approach [18, 19].

## Results

### Phase 1: expert review of translation equivalence and cultural appropriateness

Expert agreement supported the translated versions. For the QLQ-C30, item-level agreement indices ranged from 0.83 to 1.00 and the scale-level average was 0.90. For the QLQ-BR45, item-level indices ranged from 0.67 to 1.00. Lower agreement identified translation-level issues requiring review rather than item deletion or conceptual modification.

Expert comments focused mainly on wording in sensitive or clinically specific items, including QLQ-BR45 items 41, 50, 51, 53, and 57 and QLQ-C30 item 23. These items were linguistically refined without changing their constructs, item content, or standard EORTC scoring structure.

### Phase 2: cognitive debriefing

Cognitive debriefing indicated good overall acceptability. Nine of ten participants reported no difficulties; one commented on wording in three items. Minor translation-tone and orthographic issues were reviewed and corrected where consistent with the source concepts. No conceptual changes were introduced.

### Phase 3: psychometric evaluation

At baseline, 142 women participated and 47 completed the six-month follow-up (Table 3). The baseline mean age was 56.1 years; 57.7% had higher education and 76.1% were married or in a relationship. Right- and left-sided tumours were similarly represented. Baseline clinical characteristics and the health status of the follow-up subsample are summarised in Tables 4 and 5.

**Table 3:** Demographic characteristics of the study participants

Patient characteristics	Baseline (T1) *N* = 142	Follow-up (T2) *N* = 47
Age (years), mean ± SD	56.1 ± 12.8	53.7 ± 12.5
Place of residence, n (%)		
Capital region	72 (50.7)	22 (46.8)
Other regions	70 (49.3)	25 (53.2)
Nationality, n (%)		
Latvian	104 (73.2)	36 (76.6)
Russian	25 (17.6)	7 (14.9)
Other	9 (6.3)	2 (4.3)
Prefer not to answer	4 (2.8)	2 (4.3)
Level of education, n (%)		
Primary education	4 (2.8)	1 (2.1)
Secondary education	19 (13.4)	4 (8.5)
Vocational education	37 (26.1)	15 (31.9)
Higher education	82 (57.7)	27 (57.4)
Marital status, n (%)		
Single	8 (5.6)	1 (2.1)
Married/in a relationship	108 (76.1)	39 (83.0)
Divorced	13 (9.2)	5 (10.6)
Widowed	12 (8.3)	2 (4.3)
Prefer not to answer	1 (0.7)	0 (0.0)
Laterality, n (%)		
Right	72 (50.7)	24 (51.1)
Left	64 (45.1)	21 (44.7)
Both	6 (4.2)	2 (4.3)
BMI, mean ± SD	27.7 ± 5.4	27.6 ± 4.9

**Table 4:** Summary of health status, cancer characteristics, and treatment in the baseline sample at T1

Domain	Characteristic	*n*/*N* (%) or M (SD)
Data availability	Health-status questionnaire records available	142/142 (100.0%)
	Dedicated treatment-strategy records available	85/142 (59.9%)
	Clinical records available	137/142 (96.5%)
Health status	Tumor laterality: right/left/bilateral	72 (50.7%)/64 (45.1%)/6 (4.2%)
	BMI, kg/m², M (SD); median [range]	27.66 (5.37); 27.5 [17.9–44.8]
	BMI category: underweight/normal/overweight/obesity	1 (0.7%)/50 (35.5%)/47 (33.3%)/43 (30.5%)
	Any comorbid/medical condition	112 (78.9%)
	Number of comorbid conditions: 0/1/2/3/>=4	30 (21.1%)/37 (26.1%)/33 (23.2%)/13 (9.2%)/29 (20.4%)
Most frequent comorbidities	Back pain	70 (49.3%)
	High blood pressure	47 (33.1%)
	Other medical problems	32 (22.5%)
	Cardiovascular disease	20 (14.1%)
	Osteoarthritis/degenerative arthritis	19 (13.4%)
	Gastric ulcer/gastrointestinal disease	17 (12.0%)
	Lung disease	16 (11.3%)
Clinical characteristics	Menopausal status: premenopausal/postmenopausal	56 (40.9%) /81 (59.1%)
	Tumour behaviour: invasive/in situ/other	120 (87.6%)/16 (11.7%)/1 (0.7%)
	Morphology: ductal/lobular/other	117 (85.4%)/11 (8.0%)/9 (6.6%)
	Grade: 1/2/3/not applicable	24 (17.5%)/64 (46.7%)/32 (23.4%)/17 (12.4%)
	Receptor status: ER+/PR+/HER2+/triple negative	100 (73.0%)/94 (68.6%)/15 (10.9%)/24 (17.5%)
	Oncotype test performed	39 (28.5%)
	Clinical tumour size: Tis/T1/T2/T3–T4	15 (10.9%)/67 (48.9%)/38 (27.7%)/17 (12.4%)
	Clinical nodal status: N0/N1/N2-N3/Nx	89 (65.0%)/33 (24.1%)/11 (8.0%)/4 (2.9%)
	Clinical metastasis status: M0/M1/Mx	130 (94.9%)/4 (2.9%)/3 (2.2%)
MDT assigned treatment strategy	Surgical treatment	119 (86.9%)
	Radiotherapy	98 (71.5%)
	Hormonal therapy	87 (63.5%)
	Neoadjuvant/adjuvant chemotherapy	42 (30.7%)/ 18 (13.1%)
	Targeted therapy/immunotherapy	25 (18.2%)/24 (17.5%)
	Supportive therapy/palliative care	4 (2.9%)
Surgery type	Breast-conserving surgery ± mammoplasty	72/119 (60.5%)
	Mastectomy ± immediate reconstruction	47/119 (39.5%)
Documented treatment	Received MDT-recommended therapy	104/137 (75.9%)
	Sentinel lymph node biopsy/axillary dissection	113 (95.0%)/ 32 (26.9%)
	Radiotherapy	
Hormone therapy received	98 (71.5%) /89 (65.0%)	
	Neoadjuvant/adjuvant chemotherapy received	40 (29.2%)/ 18 (13.1%)
	Targeted therapy/immunotherapy received	29 (21.2%)/ 23 (16.8%)

Baseline health-status data were available for 142 participants. Clinical characteristics were based on available electronic health records; denominators therefore varied across variables. BMI categories use *n* = 141 because one implausible BMI value was excluded. Comorbidity rows list the most frequent conditions (≥ 10%). Multiple treatments could be recorded. BCS = breast-conserving surgery; MDT = multidisciplinary team

Clinical records were available for most baseline participants, although completeness varied by variable. Most recorded tumours were invasive and hormone-receptor positive, and surgery was the most commonly documented treatment, followed by radiotherapy and endocrine therapy (Table 4). The 47-person follow-up subsample was broadly similar to the baseline cohort on the main recorded health characteristics (Table 5).

**Table 5:** Summary of health status in the six-month follow-up subsample

Domain	Characteristic	*n*/*N* (%) or M (SD)
Data availability	Second-measurement participants	47/47 (100.0%)
	Health-status questionnaire records available	47/47 (100.0%)
	Treatment-strategy records available	40/47 (85.1%)
	Clinical records available	28/47 (59.6%)
Health status	Tumor laterality: right/left/bilateral	24 (51.1%)/21 (44.7%)/2 (4.3%)
	BMI, kg/m², M (SD); median [range]	27.57 (4.93); 27.0 [19.5–43.7]
	BMI category: normal/overweight/obesity	14 (29.8%)/21 (44.7%)/12 (25.5%)
	Any comorbid/medical condition	33 (70.2%)
	Number of comorbid conditions: 0/1/2/3/>=4	14 (29.8%)/11 (23.4%)/10 (21.3%)/4 (8.5%)/8 (17.0%)
Most frequent comorbidities	Back pain	23 (48.9%)
	High blood pressure	11 (23.4%)
	Other medical problems	10 (21.3%)
	Lung disease	6 (12.8%)
	Gastric ulcer/gastrointestinal disease	5 (10.6%)
	Osteoarthritis/degenerative arthritis	5 (10.6%)

The follow-up subsample included 47 participants with second-measurement data. Baseline health-status data were available for all 47; percentages are based on the available denominator for each characteristic. Comorbidity rows list the most frequent conditions (≥ 10%)

Item-level results and reliability estimates are shown in Table 6. Several QLQ-C30 scales had lower internal consistency at baseline, particularly Social Functioning, Fatigue, and Pain, whereas follow-up estimates were generally higher. In the QLQ-BR45, Body Image and Sexual Functioning showed strong internal consistency at both assessments. Estimates for conditional scales were based on smaller valid samples and should be interpreted cautiously.

**Table 6:** Item-level psychometric indicators for the EORTC QLQ-C30 and EORTC QLQ-BR45

Scale	Item	Baseline (T1)						Follow-up assessment (T2)					
		*n*	M	SD	r_it	α	ω	*n*	M	SD	r_it	α	ω
EORTC QLQ-C30													
Global health status/QoL													
Global health status/QoL (QL2)	QL2_29 general health	142	5.34	1.184	0.722	0.837	–	47	5.17	1.239	0.893	0.937	–
	QL2_30 general quality of life	142	5.12	1.114	0.722			47	5.06	1.051	0.893		
Functional scales													
Physical functioning (PF2)	PF2_1 carrying heavy objects	142	1.98	0.879	0.448	0.756	0.752	47	2.38	0.848	0.515	0.789	0.830
	PF2_2 long walks	142	1.95	0.688	0.561			47	1.96	0.833	0.758		
	PF2_3 short walks	142	2.06	0.877	0.452			47	1.36	0.605	0.748		
	PF2_4 time spent lying down or sitting	142	1.70	0.744	0.692			47	1.64	0.870	0.648		
	PF2_5 help with self-care	142	1.45	0.710	0.507			47	1.11	0.429	0.221		
Role functioning (RF2)	RF2_6 work on daily activities	142	1.25	0.497	0.501	0.541	–	47	1.70	0.623	0.699	0.817	–
	RF2_7 hobbies	142	1.04	0.220	0.501			47	1.68	0.726	0.699		
Emotional functioning (EF)	EF_21 tension	142	1.42	0.737	0.423	0.632	0.695	47	1.83	0.637	0.636	0.813	0.828
	EF_22 worry	142	1.16	0.456	0.611			47	1.98	0.571	0.585		
	EF_23 irritability	142	1.30	0.581	0.569			47	2.02	0.847	0.703		
	EF_24 depression	142	1.04	0.185	0.151			47	1.94	0.734	0.649		
Cognitive functioning (CF)	CF_20 concentration difficulties	142	1.50	0.722	0.482	0.646	–	47	1.47	0.747	0.634	0.774	–
	CF_25 memory problems	142	1.42	0.622	0.482			47	1.87	0.679	0.634		
Social functioning (SF)	SF_26 family life	142	1.39	0.641	0.174	0.291	–	47	1.81	0.798	0.720	0.831	–
	SF_27 social activities	142	1.28	0.524	0.174			47	1.96	0.932	0.720		
Symptom scales/items													
Fatigue (FA)	FA_10 need to rest	142	1.96	0.683	0.479	0.530	0.596	47	2.26	0.675	0.732	0.872	0.878
	FA_12 weakness	142	1.63	0.856	0.369			47	2.15	0.780	0.734		
	FA_18 fatigue	142	1.73	0.640	0.213			47	2.34	0.788	0.810		
Nausea and vomiting (NV)	NV_14 nausea	142	1.92	0.719	0.620	0.764	–	47	1.40	0.614	0.566	0.627	–
	NV_15 vomiting	142	2.38	0.788	0.620			47	1.11	0.312	0.566		
Pain (PA)	PA_9 pain	142	1.21	0.517	0.282	0.432	–	47	2.11	0.729	0.734	0.846	–
	PA_19 pain interferes with daily activities	142	1.39	0.641	0.282			47	1.81	0.770	0.734		
Dyspnoea (DY)	DY_8 dyspnoea	142	1.30	0.556	–	–	–	47	1.47	0.718	–	–	–
Insomnia (SL)	SL_11 insomnia	142	1.46	0.670	–	–	–	47	2.17	0.816	–	–	–
Appetite loss (AP)	AP_13 appetite loss	142	1.21	0.544	–	–	–	47	1.51	0.688	–	–	–
Constipation (CO)	CO_16 constipation	142	1.83	0.714	–	–	–	47	1.66	0.788	–	–	–
Diarrhoea (DI)	DI_17 diarrhoea	142	1.82	0.708	–	–	–	47	1.30	0.587	–	–	–
Financial difficulties (FI)	FI_28 financial difficulties	142	1.88	0.709	–	–	–	47	1.68	0.837	–	–	–
EORTC QLQ-BR45													
Functional scales													
Body Image (BI)	BI_39 physical attractiveness	140	1.43	0.701	0.726	0.865	0.859	46	1.98	1.000	0.841	0.924	0.924
	BI_40 femininity	140	1.45	0.692	0.734			46	2.02	1.064	0.863		
	BI_41 naked appearance	140	1.42	0.720	0.724			46	1.87	0.934	0.831		
	BI_42 body satisfaction	140	1.56	0.681	0.673			46	2.11	0.900	0.768		
Future Perspective (FU)	FU_43 future perspective	140	2.99	0.818	–	–	–	46	2.65	0.948	–	–	–
Sexual Functioning (SX)	SX_44 interest in sex	140	3.31	0.658	0.687	0.814	–	46	3.37	0.679	0.806	0.893	–
	SX_45 sexual activity	140	3.31	0.645	0.687			46	3.33	0.668	0.806		
Sexual Enjoyment (SE)	SE_46 sexual enjoyment	83	2.70	0.694	–	–	–	26	2.54	0.706	–	–	–
Symptom scales													
Systemic Therapy Side Effects (SYS)	SYS_31 dry mouth	140	1.66	0.716	0.437	0.690	0.687	46	1.91	0.890	0.208	0.701	0.726
	SYS_32 changes in taste	140	1.10	0.324	0.446			46	1.50	0.810	0.666		
	SYS_33 eye discomfort	140	1.48	0.605	0.452			46	1.74	0.801	0.483		
	SYS_34 hair loss	140	1.42	0.720	0.478			46	2.09	1.226	0.311		
	SYS_36 feeling unwell	140	1.53	0.662	0.415			46	1.91	0.865	0.663		
	SYS_37 hot flushes	140	1.64	0.732	0.296			46	2.50	1.188	0.457		
	SYS_38 headache	140	1.66	0.676	0.374			46	1.65	0.674	0.215		
Upset by Hair Loss (HU)	HU_35 upset by hair loss	44	2.25	0.967	–	–	–	25	2.56	1.083	–	–	–
Arm Symptoms (ARM)	ARM_47 pain in the arm or shoulder	140	1.64	0.798	0.537	0.573	0.768	46	1.98	0.802	0.554	0.744	0.747
	ARM_48 swelling in the arm or shoulder	140	1.12	0.328	0.258			46	1.46	0.721	0.547		
	ARM_49 difficulty lifting or moving the arm	140	1.34	0.609	0.472			46	1.85	0.788	0.611		
Breast Symptoms (BR)	BR_50 breast pain	140	1.71	0.662	0.527	0.693	0.728	46	1.91	0.661	0.668	0.777	0.779
	BR_51 skin swelling in the breast area	140	1.40	0.655	0.541			46	1.54	0.690	0.497		
	BR_52 skin sensitivity in the breast area	140	1.71	0.742	0.618			46	1.87	0.687	0.582		
	BR_53 skin problems in the breast area	140	1.21	0.506	0.240			46	1.65	0.706	0.582		
Endocrine Therapy Symptoms (ET)	ET_54 sweating	12	2.92	0.996	0.352	0.857	***	12	2.42	0.900	0.492	0.717	***
	ET_55 mood swings	12	2.17	0.718	0.629			12	2.25	0.965	0.351		
	ET_56 dizziness	12	2.00	0.953	0.591			12	1.83	0.835	0.542		
	ET_63 joint problems	12	2.50	0.522	0.610			12	2.17	0.389	0.111		
	ET_64 joint stiffness	12	2.42	0.793	0.930			12	2.08	0.669	0.338		
	ET_65 joint pain	12	2.42	0.793	0.903			12	2.25	0.452	0.306		
	ET_66 bone pain	12	2.00	1.044	0.942			12	2.25	0.965	0.638		
	ET_67 muscle pain	12	2.25	0.866	0.649			12	1.92	0.669	0.387		
	ET_68 weight gain	12	2.33	0.651	0.276			12	2.50	0.674	0.268		
	ET_69 problems caused by weight gain	12	1.92	0.996	0.050			12	2.50	1.168	0.334		
Skin/Mucosis Symptoms (SM)	SM_57 mouth pain	140	1.06	0.299	0.339	0.675	***	46	1.13	0.400	0.186	0.621	***
	SM_58 redness in the mouth	140	1.07	0.332	0.292			46	1.07	0.250	0.286		
	SM_59 pain in the palms and soles	140	1.29	0.566	0.556			46	1.61	0.682	0.294		
	SM_60 redness/rash	140	1.02	0.188	0.049			46	1.09	0.285	0.128		
	SM_61 tingling in the fingers and toes	140	1.56	0.779	0.623			46	2.00	0.989	0.734		
	SM_62 numbness in the fingers or toes	140	1.24	0.583	0.585			46	1.57	0.779	0.580		
Endocrine Sexual Symptoms (ES)	ES_70 vaginal dryness	83	1.31	0.623	0.584	0.740	0.748	26	1.88	0.952	0.659	0.887	0.875
	ES_71 vaginal discomfort	83	1.20	0.406	0.645			26	1.73	0.827	0.840		
	ES_72 vaginal pain during sexual activity	83	1.17	0.377	0.378			26	1.77	0.765	0.815		
	ES_73 vaginal dryness during sex	83	1.27	0.520	0.593			26	1.85	0.784	0.733		

M = mean; SD = standard deviation; r_it = corrected item–total correlation; α = Cronbach’s alpha; ω = McDonald’s omega. Valid n indicates participants with valid item/scale data at the respective assessment. QLQ-C30 global health/QoL items range from 1 to 7; other EORTC item responses range from 1 to 4. QLQ-BR45 items 44–46 are shown after reverse coding, consistent with scoring of Sexual Functioning and Sexual Enjoyment. Single-item scales are descriptive only. Conditional QLQ-BR45 scales were structurally missing when not applicable. *** Omega was not reported where assumptions for a stable estimate were not met

Multitrait scaling results are summarised in Table 7. The strongest scaling support was observed for QLQ-BR45 Body Image, Sexual Functioning, and Breast Symptoms, with all items showing scaling success. Endocrine Sexual Symptoms also performed relatively well, although based on a smaller valid sample. Scaling success was weaker for several QLQ-C30 domains and for QLQ-BR45 Systemic Therapy Side Effects, Arm Symptoms, Endocrine Therapy Symptoms, and Skin/Mucosis Symptoms, indicating overlap among clinically related functioning and symptom constructs.

**Table 7:** Multitrait Scaling Summary for the EORTC QLQ-C30 and EORTC QLQ-BR45 at Baseline

Instrument	Scale	k	*N*	Item–own *r*	Highest item–other |*r*|	Convergent success	Scaling success
C30	Global health/QoL (QL2)	2	142	0.72	0.60–0.62	2/2	2/2 (100%)
C30	Physical functioning (PF2)	5	142	0.45–0.69	0.41–0.68	5/5	3/5 (60%)
C30	Role functioning (RF2)	2	142	0.50	0.42–0.52	2/2	1/2 (50%)
C30	Emotional functioning (EF)	4	142	0.15–0.61	0.25–0.64	3/4	0/4 (0%)
C30	Cognitive functioning (CF)	2	142	0.48	0.65–0.70	2/2	0/2 (0%)
C30	Social functioning (SF)	2	142	0.17	0.41–0.69	0/2	0/2 (0%)
C30	Fatigue (FA)	3	142	0.21–0.48	0.36–0.70	1/3	0/3 (0%)
C30	Nausea/vomiting (NV)	2	142	0.62	0.24–0.36	2/2	2/2 (100%)
C30	Pain (PA)	2	142	0.28	0.50–0.70	0/2	0/2 (0%)
BR45	Body image (BI)	4	140	0.67–0.73	0.39–0.44	4/4	4/4 (100%)
BR45	Sexual functioning (SX)	2	140	0.69	0.33–0.44	2/2	2/2 (100%)
BR45	Systemic side effects (SYS)	7	140	0.30–0.48	0.39–0.78	5/7	2/7 (29%)
BR45	Arm symptoms (ARM)	3	140	0.26–0.54	0.31–0.71	2/3	1/3 (33%)
BR45	Breast symptoms (BR)	4	140	0.24–0.62	0.18–0.45	3/4	4/4 (100%)
BR45	Endocrine therapy symptoms (ET)	10	12	0.05–0.94	0.39–0.88	7/10	4/10 (40%)
BR45	Skin/mucosis symptoms (SM)	6	140	0.05–0.62	0.14–0.47	3/6	3/6 (50%)
BR45	Endocrine sexual symptoms (ES)	4	83	0.38–0.65	0.12–0.84	3/4	3/4 (75%)

T1 = baseline; k = number of items. Single-item scales were excluded. Item–own correlations are corrected item–total correlations. Highest item–other |r| is the highest absolute correlation with another multi-item scale within the same questionnaire/module. Convergent success = item–own *r* ≥ .40; scaling success = item–own r exceeding the highest item–other |r|. Conditional QLQ-BR45 scales used available valid subsamples

Standardised scale scores are presented in Table 8. Descriptively, global health/QoL was relatively stable at follow-up and physical functioning changed little, whereas role, emotional, cognitive, and social functioning were lower. Several treatment-related symptom scores were higher at follow-up. Conditional QLQ-BR45 scales had reduced valid N.

**Table 8:** Descriptive Statistics for the EORTC QLQ-C30, EORTC QLQ-BR45, and Stress Symptom Scale Scores at Baseline and Follow-Up

Scale	*N* of items	Valid *N*	Baseline (T1)					Follow-up assessment (T2)			
			M	SD	Min	Max	Valid *N*	M	SD	Min	Max
EORTC QLQ-C30											
Global health status/QoL											
Global health status/QoL (QL2)	2	142	70.48	17.77	16.67	100.00	47	68.62	18.57	33.33	100.00
Functional scales											
Physical functioning (PF2)	5	142	72.39	18.58	0.00	100.00	47	77.02	18.11	20.00	100.00
Role functioning (RF2)	2	142	95.19	10.61	16.67	100.00	47	76.95	20.72	33.33	100.00
Emotional functioning (EF)	4	142	92.37	12.18	41.67	100.00	47	68.62	18.81	16.67	100.00
Cognitive functioning (CF)	2	142	84.74	19.30	0.00	100.00	47	77.66	21.50	16.67	100.00
Social functioning (SF)	2	142	88.73	14.93	33.33	100.00	47	70.57	26.74	0.00	100.00
Symptom scales/items											
Fatigue (FA)	3	142	25.74	17.52	0.00	77.78	47	41.61	22.28	0.00	88.89
Nausea and vomiting (NV)	2	142	38.26	22.61	0.00	100.00	47	8.51	13.85	0.00	50.00
Pain (PA)	2	142	10.09	15.50	0.00	83.33	47	31.91	23.27	0.00	83.33
Dyspnoea (DY)	1	142	9.86	18.53	0.00	100.00	47	15.60	23.93	0.00	66.67
Insomnia (SL)	1	142	15.26	22.32	0.00	100.00	47	39.01	27.20	0.00	100.00
Appetite loss (AP)	1	142	7.04	18.12	0.00	100.00	47	17.02	22.92	0.00	66.67
Constipation (CO)	1	142	27.70	23.81	0.00	100.00	47	21.99	26.26	0.00	100.00
Diarrhoea (DI)	1	142	27.46	23.59	0.00	100.00	47	9.93	19.55	0.00	66.67
Financial difficulties (FI)	1	142	29.34	23.65	0.00	100.00	47	22.70	27.89	0.00	100.00
EORTC QLQ-BR45											
Functional scales											
Body Image (BI)	4	140	84.52	19.65	0.00	100.00	46	66.85	29.37	0.00	100.00
Future Perspective (FU)	1	140	33.57	27.26	0.00	100.00	46	44.93	31.60	0.00	100.00
Sexual Functioning (SX)	2	140	22.98	19.94	0.00	66.67	46	21.74	21.33	0.00	66.67
Sexual Enjoyment (SE)	1	83	43.37	23.13	0.00	100.00	26	48.72	23.53	0.00	100.00
Symptom scales											
Systemic Therapy Side Effects (SYS)	7	140	16.60	12.77	0.00	61.90	46	30.02	18.78	0.00	71.43
Upset by Hair Loss (HU)	1	44	41.67	32.25	0.00	100.00	25	52.00	36.11	0.00	100.00
Arm Symptoms (ARM)	3	140	12.22	14.92	0.00	66.67	46	25.36	20.91	0.00	100.00
Breast Symptoms (BR)	4	140	16.96	15.56	0.00	66.67	46	24.82	17.70	0.00	75.00
Endocrine Therapy Symptoms (ET)	10	12	43.06	18.72	20.00	83.33	12	40.56	14.20	16.67	63.33
Skin/Mucosis Symptoms (SM)	6	140	6.90	10.30	0.00	50.00	46	13.65	12.27	0.00	50.00
Endocrine Sexual Symptoms (ES)	4	83	7.93	12.27	0.00	50.00	26	26.92	24.07	0.00	100.00
Stress Symptom Scale											
Physical Stress Symptoms (F)	21	141	1.01	0.70	0.00	4.57	43	1.23	0.71	0.24	2.86
Impaired Cognitive Functioning (K)	14	141	1.02	0.85	0.00	4.86	43	1.33	0.92	0.00	3.93
Psychological Stress Symptoms (P)	15	141	0.75	0.85	0.00	4.67	43	0.88	0.85	0.00	3.07
Emotional Stress Symptoms (E)	6	141	0.77	0.74	0.00	3.50	43	1.08	0.92	0.00	3.50
Stress symptoms sum (SSS)	56	141	3.55	2.74	0.00	17.60	43	4.51	2.96	0.59	12.89

EORTC scores range from 0 to 100. Higher functioning/global health scores indicate better functioning or quality of life; higher symptom scores indicate greater symptom burden. Stress Symptom Scale domains are means from 0 to 5; SSS is the sum of the four domain means. Valid N indicates available scale scores. Conditional QLQ-BR45 scales were structurally missing when not applicable; valid N therefore varies across scales

**Table 9:** Paired baseline and six-month follow-up comparisons (Wilcoxon Signed-Rank Test)

Scale	*n*	T1 M (SD)	T2 M (SD)	ΔM	W	*p*
EORTC QLQ-C30						
Global health status/QoL (QL2)	47	71.45 (16.09)	68.62 (18.57)	− 2.84	292.0	0.368
Physical functioning (PF2)	47	76.31 (14.70)	77.02 (18.11)	0.71	392.0	0.617
Role functioning (RF)	47	97.87 (5.62)	76.95 (20.72)	− 20.92	4.0	< 0.001***
Emotional functioning (EF)	47	93.79 (10.05)	68.62 (18.81)	− 25.18	5.0	< 0.001***
Cognitive functioning (CF)	47	87.59 (14.52)	77.66 (21.50)	− 9.93	119.5	0.006**
Social functioning (SF)	47	89.72 (12.31)	70.57 (26.74)	− 19.15	37.5	< 0.001***
Fatigue (FA)	47	23.40 (17.22)	41.61 (22.28)	18.2	48.5	< 0.001***
Nausea and vomiting (NV)	47	37.94 (20.17)	8.51 (13.85)	− 29.43	46.5	< 0.001***
Pain (PA)	47	7.09 (11.39)	31.91 (23.27)	24.82	32.0	< 0.001***
Dyspnoea (DY)	47	7.80 (18.67)	15.60 (23.93)	7.8	63.5	0.056
Insomnia (SL)	47	12.06 (17.62)	39.01 (27.20)	26.95	22.0	< 0.001***
Appetite loss (AP)	47	7.09 (13.79)	17.02 (22.92)	9.93	57.0	0.016*
Constipation (CO)	47	29.08 (22.65)	21.99 (26.26)	− 7.09	155.5	0.150
Diarrhoea (DI)	47	30.50 (20.65)	9.93 (19.55)	− 20.57	54.0	< 0.001***
Financial difficulties (FI)	47	28.37 (19.63)	22.70 (27.89)	− 5.67	200.5	0.311
EORTC QLQ-BR45						
Body Image (BI)	46	89.67 (14.93)	66.85 (29.37)	− 22.83	28.5	< 0.001***
Future Perspective (FU)	46	31.16 (25.73)	44.93 (31.60)	13.77	55.5	0.004**
Sexual Functioning (SX)	46	27.17 (19.68)	21.74 (21.33)	− 5.43	90.5	0.084
Sexual Enjoyment (SE)	23	46.38 (16.63)	49.28 (24.35)	2.9	19.0	0.662
Systemic Therapy Side Effects (SYS)	46	14.80 (9.90)	30.02 (18.78)	15.22	74.0	< 0.001***
Upset by Hair Loss (HU)	12	50.00 (30.15)	63.89 (26.43)	13.89	0.0	0.062
Arm Symptoms (ARM)	46	7.97 (12.32)	25.36 (20.91)	17.39	13.5	< 0.001***
Breast Symptoms (BR)	46	15.76 (16.12)	24.82 (17.70)	9.06	195.0	0.011*
Endocrine Therapy Symptoms (ET)	3	42.22 (10.18)	42.22 (16.44)	− 0.0	3.0	1.000
Skin/Mucosis Symptoms (SM)	46	4.23 (6.33)	13.65 (12.27)	9.42	51.0	< 0.001***
Endocrine Sexual Symptoms (ES)	23	11.59 (12.75)	28.62 (24.08)	17.03	0.0	< 0.001***
Stress Symptom Scale						
Physical Stress Symptoms (F)	43	0.91 (0.49)	1.23 (0.71)	0.31	219.0	0.006**
Impaired Cognitive Functioning (K)	43	0.92 (0.68)	1.33 (0.92)	0.41	250.5	0.020*
Psychological Stress Symptoms (P)	43	0.61 (0.63)	0.88 (0.85)	0.27	277.5	0.075
Emotional Stress Symptoms (E)	43	0.74 (0.76)	1.08 (0.92)	0.34	185.5	0.034*
Stress symptoms sum (SSS)	43	3.19 (2.16)	4.51 (2.96)	1.33	246.5	0.006**

Analyses include participants with valid paired T1 and T2 scores for each scale. Positive ΔM indicates higher follow-up scores. Higher EORTC functioning scores indicate better functioning; higher symptom scores indicate greater burden. Wilcoxon signed-rank tests were used because of the small follow-up sample and reduced valid n for conditional scales. Endocrine Therapy Symptoms should be interpreted with particular caution because of the very small paired sample

*p < 0.05. **p < 0.01. ***p < 0.001

Paired analyses (Table 9) showed significant decreases in role, emotional, cognitive, and social functioning and increases in fatigue, pain, insomnia, and appetite loss. QLQ-BR45 Body Image decreased, Future Perspective increased, and several treatment-related symptom scales increased. Physical, cognitive, emotional, and total stress symptom scores also increased. These clinically plausible changes are preliminary because follow-up involved only 47 participants and some conditional scales had substantially smaller paired samples.

**Table 10:** Inter-scale correlations between the EORTC QLQ-C30 and EORTC QLQ-BR45 scales at baseline

	PF2	RF2	EF	CF	SF	FA	NV	PA	DY	SL	AP	CO	DI	FI	QL2
BI	0.493***	0.285***	0.333***	0.428***	0.391***	− 0.484***	− 0.372***	− 0.418***	− 0.165	− 0.354***	− 0.234**	− 0.364***	− 0.217**	− 0.371***	0.426***
FU	0.271**	0.110	0.182*	0.216*	0.205*	− 0.277***	− 0.509***	− 0.237**	− 0.039	− 0.193*	− 0.189*	− 0.429***	− 0.419***	− 0.306***	0.288***
SX	0.137	0.033	0.127	0.088	0.120	− 0.207*	− 0.123	− 0.061	0.010	− 0.248**	− 0.072	− 0.165	− 0.116	− 0.140	0.212*
SE	0.163	0.153	0.112	0.080	0.015	− 0.148	− 0.141	− 0.286**	− 0.141	− 0.154	− 0.138	− 0.248*	− 0.244*	− 0.217*	0.354**
SYS	− 0.501***	− 0.319***	− 0.292***	− 0.286***	− 0.333***	0.476***	0.238**	0.371***	0.091	0.288***	0.243**	0.228**	0.179*	0.570***	− 0.495***
HU	− 0.069	0.078	− 0.151	− 0.009	− 0.230	0.159	0.182	0.202	− 0.090	0.035	0.194	− 0.053	0.023	0.036	− 0.166
ARM	− 0.395***	− 0.284***	− 0.448***	− 0.349***	− 0.364***	0.453***	0.121	0.367***	0.191*	0.484***	0.153	0.122	0.065	0.370***	− 0.406***
BR	− 0.305***	− 0.237**	− 0.259**	− 0.370***	− 0.273**	0.249**	0.168*	0.275***	0.211*	0.362***	0.185*	0.097	0.041	0.289***	− 0.238**
ET	− 0.819**	− 0.333	− 0.843***	− 0.343	− 0.197	0.684*	0.199	0.347	− 0.299	− 0.062	0.122	0.178	0.479	0.829***	− 0.510
SM	− 0.367***	− 0.228**	− 0.312***	− 0.392***	− 0.286***	0.422***	0.114	0.385***	0.127	0.302***	0.120	0.255**	0.169*	0.388***	− 0.458***
ES	0.041	0.010	0.015	− 0.065	0.005	− 0.012	0.145	0.166	0.040	− 0.054	0.062	0.074	0.084	0.042	− 0.029

*p < 0.05, **p < 0.01, ***p < 0.001

Correlations used pairwise deletion, so valid N varied across coefficients. Scale-specific valid N values are presented in Table 8 and should be considered for conditional QLQ-BR45 scales

Baseline inter-scale correlations (Table 10) were broadly consistent with the expected scoring directions: functioning domains tended to correlate positively with other functioning domains and negatively with symptom domains, while symptom domains tended to correlate positively with one another. The pattern provides partial support for construct validity and scale distinctiveness.

**Table 11:** Correlations between the EORTC QLQ-C30, EORTC QLQ-BR45, and stress symptom scale scores at baseline

QLQ-C30	F	K	*P*	E	SSS
PF2	− 0.525***	− 0.377***	− 0.252**	− 0.174*	− 0.386***
RF2	− 0.339***	− 0.234**	− 0.131	− 0.071	− 0.226**
EF	− 0.261**	− 0.240**	− 0.113	− 0.098	− 0.218**
CF	− 0.358***	− 0.246**	− 0.167*	− 0.168*	− 0.262**
SF	− 0.298***	− 0.170*	− 0.105	− 0.061	− 0.178*
FA	0.429***	0.352***	0.242**	0.117	0.333***
NV	0.406***	0.498***	0.483***	0.483***	0.545***
PA	0.386***	0.262**	0.160	0.246**	0.299***
DY	0.132	0.102	0.022	0.055	0.077
SL	0.298***	0.159	0.061	0.021	0.160
AP	0.302***	0.388***	0.279***	0.234**	0.352***
CO	0.406***	0.460***	0.395***	0.579***	0.521***
DI	0.388***	0.478***	0.572***	0.505***	0.565***
FI	0.482***	0.272**	0.228**	0.169*	0.338***
QL2	− 0.519***	− 0.419***	− 0.269**	− 0.268**	− 0.433***

QLQ-BR45	F	K	*P*	E	SSS_sum
BI	− 0.447***	− 0.437***	− 0.393***	− 0.322***	− 0.469***
FU	− 0.346***	− 0.323***	− 0.299***	− 0.347***	− 0.377***
SX	− 0.076	− 0.061	− 0.066	− 0.118	− 0.114
SE	− 0.416***	− 0.283**	− 0.292**	− 0.342**	− 0.369***
SYS	0.707***	0.368***	0.212*	0.249**	0.438***
HU	0.093	0.093	− 0.022	− 0.047	0.061
ARM	0.417***	0.146	0.074	0.127	0.221**
BR	0.226**	0.118	0.144	0.114	0.162
ET	0.639*	0.544	0.428	0.264	0.462
SM	0.534***	0.228**	0.185*	0.276**	0.342***
ES	0.090	0.105	− 0.109	− 0.048	0.005

*p < 0.05, **p < 0.01, ***p < 0.001

Correlations used pairwise deletion, so valid N varied across coefficients. Scale-specific valid N values are presented in Table 8 and should be considered for conditional QLQ-BR45 scales

Correlations with the Stress Symptom Scale (Table 11) were also generally in the hypothesised directions. Most QLQ-C30 functioning scales correlated negatively with stress symptoms, whereas symptom scales generally correlated positively. QLQ-BR45 functioning scales showed mainly negative and several symptom scales positive associations. Weaker or non-significant associations were observed for some treatment-related and conditional domains, which were less clinically salient before treatment or had smaller valid samples.

## Discussion

This study provides the first cross-cultural adaptation and initial psychometric evaluation of the Latvian EORTC QLQ-C30 and QLQ-BR45 in women with breast cancer. Expert review and cognitive debriefing supported the linguistic, conceptual, and cultural appropriateness of the translations, while psychometric analyses provided preliminary support for several scales. The findings should be interpreted as initial validation evidence rather than confirmation of all measurement properties.

The expert review was deliberately framed as an assessment of translation equivalence and cultural appropriateness, not a re-evaluation of the established content validity of the original EORTC instruments. Translation-level refinements were most relevant for sensitive domains such as body image and sexuality, where wording can carry culturally specific meanings. Similar challenges have been reported in other QLQ-BR45 adaptations [20–22]. Importantly, no items were deleted and no constructs or scoring procedures were changed.

Reliability and multitrait scaling results were strongest for several QLQ-BR45 domains, particularly Body Image, Sexual Functioning, and Breast Symptoms. Some QLQ-C30 and QLQ-BR45 scales showed weaker internal consistency or scaling success. This may partly reflect short scales, smaller conditional subsamples, and clinical overlap among fatigue, pain, endocrine symptoms, skin/mucosal problems, arm symptoms, and functioning. Accordingly, individual reliability coefficients were interpreted together with item-scale correspondence and construct-validity evidence rather than as stand-alone proof of measurement quality [23, 24].

Construct-validity findings were broadly consistent with a priori expectations. Functioning and symptom scales generally correlated in theoretically plausible directions, and higher stress-related symptom burden was associated with poorer functioning and greater EORTC symptom burden. The Stress Symptom Scale is not cancer-specific and was therefore used only as a related external comparison measure, not as a criterion for cancer-specific HRQoL. Its use was justified by the limited availability of validated Latvian-language comparison measures and conceptual overlap in physical, cognitive, psychological, and emotional symptoms.

The six-month results showed clinically plausible deterioration in several functioning domains and increases in treatment-related symptoms, alongside relative stability in global health status and physical functioning. However, only 47 participants completed follow-up and several conditional scales had much smaller paired samples. These findings therefore indicate possible sensitivity to change but do not establish responsiveness. Additional longitudinal studies with better retention and adequate representation of treatment subgroups are required.

Several limitations remain. The study was conducted at a single specialised hospital using a convenience sample, and incomplete clinical/MDT information and small or unbalanced subgroups precluded robust known-groups comparisons. Attrition may have introduced selection bias, and conditional QLQ-BR45 scales reduced precision for reliability, correlations, and change analyses. The sample was also insufficient for confirmatory factor analysis or measurement invariance testing. Finally, the QLQ-BR45 was used because the final QLQ-BR42 was not available when the project began; future Latvian studies should evaluate transition to the updated module [6]. These limitations support the need for larger, multicentre studies examining internal structure, known-groups validity, measurement invariance, and responsiveness.

## Conclusion

The Latvian EORTC QLQ-C30 and QLQ-BR45 were linguistically, conceptually, and culturally appropriate and showed acceptable preliminary psychometric performance in women with breast cancer. The findings provide initial support for cautious use in Latvian oncology research and clinical practice to assess HRQoL and breast cancer-specific symptoms. Larger multicentre and longitudinal studies are needed to evaluate additional measurement properties, particularly known-groups validity, internal structure, responsiveness, and transition to the EORTC QLQ-BR42.

## Data Availability

The data set generated and analysed during the current study is stored in a publicly accessible data repository (DOI: https://doi.org/10.48510/FK2/BJWIRG). The de‑identified data are available from the corresponding author on reasonable request.

## Declarations

### Patient, public or community engagement

People with lived experience were involved in the translation evaluation through cognitive debriefing, and the breast cancer patient association “Pārdzīvot” supported recruitment for this phase. Patients were not involved as coinvestigators in the study design, statistical analysis, or dissemination planning.

## References

[1] International Agency for Research on Cancer (2024). Cancer Today [Data visualization interface]. https://gco.iarc.who.int/today/en/dataviz/pie?mode=cancer&types=0&sexes=2&populations=428

[2] Tang, W. Z., Lu, Y. Q., Zhu, S. R., Teng, Y. J., Wei, T. F., Chen, G. L., & Jia, K. (2024). Quality of life and its predictors among breast cancer patients treated with surgery—a retrospective minimum 3-year follow-up study. *Frontiers in Oncology*. 10.3389/fonc.2024.1466625 PMC11626211 39655077

[3] Holt-Kedde, I. L., Sadok, N., Krabbe-Timmerman, I. S., de Bock, G. H., Sidorenkov, G., & Werker, P. M. N. (2025). Quality of life after alloplastic versus autologous breast reconstruction: The influence of patient characteristics on outcomes. *Breast Care*, *20*(2), 95–110. 10.1159/000543677 PMC12005696 40256673

[4] European Organisation for Research and Treatment of Cancer (n.d.). Questionnaires. https://qol.eortc.org/questionnaires/

[5] Bjelic-Radisic, V., Cardoso, F., Cameron, D., Brain, E., Kuljanic, K., da Costa, R. A., Conroy, T., Inwald, E. C., Serpentini, S., Pinto, M., Weis, J., Morag, O., Lindviksmoen Astrup, G., Tomaszweksi, K. A., Pogoda, K., Sinai, P., Sprangers, M., Aaronson, N., Velikova, G., Greimel, E., Arraras, J., Bottomley, A., Bleiker, E., Bliem, B., Chie, W., Creutzberg, C., Deville, V., Duhoux, F., Eilf, K., Hartup, S., Koller, M., Nagele, E., Nicolatou-Galitis, O., Oberguggenberger, A., Schmalz, C., & Winters, Z. (2020). An international update of the EORTC questionnaire for assessing quality of life in breast cancer patients: EORTC QLQ-BR45. *Annals of Oncology*, *31*(2), 283–288. 10.1016/j.annonc.2019.10.027 31959345

[6] Bjelic-Radisic, V., Cardoso, F., Weis, J., Pogoda, K., Arraras, J. I., Greimel, E., Bottomley, A., Cameron, D., Brain, E., Hartup, S., da Costa Vieira, R. A., Hoefnagels, N., Huang, C. C., Shamieh, O., Pinto, M., Belay, Y. B., Serpentini, S., Bleiker, E., Nookala Krishnamurthy, M., Shimomura, A., Sturm-Inwald, E. C., Getu, M. A., Bliem, B., Astrup, G., Morag, O., Kikawa, Y., Kuljanic, K., Nevries, N., Sprangers, M., Aaronson, N. K., Sinai, P., Tomaszewski, K., Galalae, R., Conroy, T., Duhoux, F., Chie, W., & Velikova, G. (2025). An international Phase IV field study - psychometric properties of the updated module on assessing quality of life of patients with breast cancer EORTC QLQ-BR42. *The Breast*, *80*, 103890. 10.1016/j.breast.2025.103890 PMC11867226 39947087

[7] EORTC Quality of Life Group (n.d.–a). EORTC translation procedure. https://qol.eortc.org/manuals/

[8] Kuliś, D., Bottomley, A., Velikova, G., Greimel, E., & Koller, M. (2017). *EORTC Quality of Life Group translation procedure* (4 ed.). European Organisation for Research and Treatment of Cancer.

[9] EORTC Quality of Life Group (n.d.–b). EORTC QLQ–BR45 scoring manual. https://qol.eortc.org/questionnaires/

[10] Johnson, C., Aaronson, N., Blazeby, J. M., Bottomley, A., Fayers, P., Koller, M., Kuliś, D., Ramage, J., Sprangers, M., Velikova, G., & Young, T. (2011). *EORTC Quality of Life Group guidelines for developing questionnaire modules* (4 ed.). European Organisation for Research and Treatment of Cancer.

[11] Fayers, P. M., Aaronson, N. K., Bjordal, K., Groenvold, M., Curran, D., & Bottomley, A. (2001). *The EORTC QLQ-C30 scoring manual* (3 ed.). European Organisation for Research and Treatment of Cancer.

[12] Aaronson, N. K., Ahmedzai, S., Bergman, B., Bullinger, M., Cull, A., Duez, N. J., Filiberti, A., Flechtner, H., Fleishman, S. B., Haes, J. C. J. M., Kaasa, S., Klee, M., Osoba, D., Razavi, D., Rofe, P. B., Schraub, S., Sneeuw, K., Sullivan, M., & Takeda, F. (1993). The European Organization for research and treatment of cancer QLQ-C30: A quality-of-life instrument for use in international clinical trials in oncology. *JNCI: Journal of the National Cancer Institute*, *85*(5), 365–376. 10.1093/jnci/85.5.365 8433390

[13] EORTC Quality of Life Group [QLG] (n.d.). EORTC Quality of Life. https://qol.eortc.org/

[14] Dudkina, A., & Perepjolkina, V. (2021). Development and initial validation of the Stress Symptoms Scale. *SOCIETY INTEGRATION EDUCATION Proceedings of the International Scientific Conference*, *7*, 67–85. 10.17770/sie2021vol7.6426

[15] Cronbach, L. J. (1951). Coefficient alpha and the internal structure of tests. *Psychometrika*, *16*(3), 297–334. 10.1007/BF02310555

[16] Dunn, T. J., Baguley, T., & Brunsden, V. (2014). From alpha to omega: A practical solution to the pervasive problem of internal consistency estimation. *British Journal of Psychology*, *105*(3), 399–412. 10.1111/bjop.12046 24844115

[17] McDonald, R. P. (1999). *Test theory: A unified treatment*. Lawrence Erlbaum Associates.

[18] Lynn, M. R. (1986). Determination and quantification of content validity. *Nursing Research*, 35(6). 3640358

[19] Polit, D. F., & Beck, C. T. (2006). The content validity index: Are you sure you know what’s being reported? Critique and recommendations. *Research in Nursing & Health*, *29*(5), 489–497. 10.1002/nur.20147 16977646

[20] Ehab, B. H., Hussein, R. R. S., Abdullah, E. A., Ahmed, Z. M., & Elsherbiny, R. M. (2022). Cultural adaptation and validation of the EORTC QLQ-BR45 to assess health-related quality of life of breast cancer patients. *European Pharmaceutical Journal*, *68*(2), 41–48. 10.2478/afpuc-2021-0018

[21] Getu, M. A., Wang, P., Kantelhardt, E. J., Seife, E., Chen, C., & Addissie, A. (2022). Translation and validation of the EORTC QLQ-BR45 among Ethiopian breast cancer patients. *Scientific Reports*, *12*(1), 605. 10.1038/s41598-021-02511-9 PMC9338022 35906247

[22] Kidayi, P. L., Pakpour, A. H., Saboonchi, F., Bray, F., Manhica, H., Mtuya, C. C., Serventi, F., Aune, R. E., Mahande, M. J., & Björling, G. (2023). Cross-Cultural Adaptation and Psychometric Properties of the Swahili Version of the European Organization for Research and Treatment of Cancer (EORTC) QLQ-BR45 among Breast Cancer Patients in Tanzania. *Healthcare*, *11*, 2467. 10.3390/healthcare11182467 PMC10530899 37761665

[23] Boateng, G. O., Neilands, T. B., Frongillo, E. A., Melgar-Quiñonez, H. R., & Young, S. L. (2018). Best practices for developing and validating scales for health, social, and behavioral research: A primer. *Frontiers in Public Health*, *6*, 2018. 10.3389/fpubh.2018.00149 PMC6004510 29942800

[24] Mokkink, L B., Elsman, E. B. M., & Terwee, C. B. (2024). COSMIN guideline for systematic reviews of patient-reported outcome measures version 2.0. *Quality of Life Research*, *33*(11), 2929–2939. 10.1007/s11136-024-03761-6. PMC11541334 39198348

[25] OECD/European Observatory on Health Systems and Policies. (2019). Latvia: Country Health Profile 2019. 10.1787/4dd50c09-en

[26] Manrique-Hernandez, E. F., Goes, B., Hoyos Madera, E., Bermon, K., Hurtado-Ortiz, A., Licht-Ardila, A., M., & Nieves-Cuervo, G. M. (2024). Perspectives on quality of life in women with breast cancer. *Indian Journal of Palliative Care*, *30*(4), 347–352. 10.25259/IJPC_37_2024 PMC11618642 39650580

[27] Hasanah, U., Ahmad, M., Prihantono, P., Usman, A. N., Arsyad, A., & Agustin, D. I. (2024). The quality of life assessment of breast cancer patients. *Breast Disease*, *43*(1), 173–185. 10.3233/BD-249008 PMC11191531 38875026

[28] Vasanth, S., Lakshmana Kumar, V., Sopnajothi, S., Jeyaprakash, P., & Vijayabaskar, P. (2024). Carcinoma breast and quality of life: A prospective observational study. *Global Journal for Research Analysis*, *13*(7), 1–2. 10.36106/gjra/7904909

[29] Tadesse, D., Tadesse, F., Taye, G., Van Roey, T., Fayers, P., & Bjordal, K. (2022). Translation and validation of the EORTC QLQ–BR45 among Ethiopian breast cancer patients. *Scientific Reports*, *12*, 12786. 10.1038/s41598-022-17088-4 PMC9338022 35906247

[30] Abma, I. L., Rovers, M., & van der Wees, P. J. (2016). Appraising convergent validity of patient-reported outcome measures in systematic reviews: constructing hypotheses and interpreting outcomes. *BMC Research Notes*, *9*(1), 226. 10.1186/s13104-016-2034-2 PMC4837507 27094345

[31] Ashing-Giwa, K. T., & Lim, J. W. (2011). Examining emotional outcomes among a multiethnic cohort of breast cancer survivors. *Oncology Nursing Forum*, *38*(3), 279–288. 10.1188/11.ONF.279-288 21531679

[32] Calderon, C., Ferrando, P. J., Lorenzo-Seva, U., Ferreira, E., Lee, E. M., Oporto-Alonso, M., Obispo-Portero, B. M., Mihic-Góngora, L., Rodríguez-González, A., & Jiménez-Fonseca, P. (2022). Psychometric properties of the Spanish version of the European Organization for Research and Treatment of Cancer Quality of Life Questionnaire (EORTC QLQ-C30). *Quality of Life Research*, *31*(6), 1859–1869. 10.1007/s11136-021-03068-w PMC9098585 34928470

[33] Ministru Kabinets (2025). Par Veselības aprūpes pakalpojumu onkoloģijas jomā uzlabošanas plānu 2025.–2027. gadam. Ministru kabineta rīkojums Nr. 692. Latvijas Vēstnesis.

[34] Neve, O. M., van Benthem, P. P. G., Stiggelbout, A. M., & Hensen, E. F. (2021). Response rate of patient reported outcomes: the delivery method matters. *BMC Medical Research Methodology*, *21*(1), 220. 10.1186/s12874-021-01419-2 PMC8540148 34686129

